# Gastrointestinal failure score alone and in combination with SOFA score in the assessment of the critically ill patients

**DOI:** 10.1186/cc9929

**Published:** 2011-03-11

**Authors:** N Abed, L Mohammed, A Metwaly, M Hussien

**Affiliations:** 1 Cairo University, Cairo, Egypt; 2 Theodor Bilhars Research Institute, Cairo, Egypt

## Introduction

Gastrointestinal problems occur frequently and are associated with an adverse outcome in critically ill patients; despite this, gastrointestinal (GI) function is not included in any of the widely used scoring systems assessing organ failures in critical illness. Several studies have demonstrated an impact of intra-abdominal hypertension (IAH) on mortality [1]. With the goal of developing a scoring system for GI failure, Reintam and colleagues combined GI symptoms and IAH into a five-grade scale - the Gastrointestinal Failure Score - and tested it among critically ill patients in Estonian ICUs [2]. The aim of our study was to evaluate the GIF score in our Egyptian ICUs regarding validity and impact on mortality and comparing this with the SOFA score.

## Methods

We studied 109 mechanically ventilated patients on day 1 admitted to the general ICU of Kasr El Aini Hospital and Theodor Bilharz Research Institute in the period from March 2009 to November 2009. The SOFA + GIF scores were calculated each day by summarizing the SOFA score and the GIF score of the respective day in each patient.

## Results

FI developed in 35.8%, IAH in 26.9% and both of them together in 14.7% of all patients. Compared with patients with mean GIF = 0, patients with mean GIF higher than 0 and lower than or equal 2 and mean GIF higher than 2 show higher ICU mortality (100%, 81.4% vs. 48.2% *P *< 0.0001), respectively. The GIF score integrated into the SOFA score allowed a better prediction of ICU mortality than the SOFA score alone shown by AUC 0.92 and 0.890, respectively.

## Conclusions

The mean GIF score in the first 3 days on the ICU demonstrated a high prognostic value in prediction of ICU mortality. Further multicenter studies should confirm whether GIF score could be advocated as an adjuvant subscore for GI tract assessment in the SOFA score.
